# MET and SLFN11 as a Players in the SCLC Molecular Subtyping Game

**DOI:** 10.3390/ijms26136095

**Published:** 2025-06-25

**Authors:** Anna Grenda, Natalia Galant, Aleksandra Łomża-Łaba, Paweł Krawczyk, Tomasz Jankowski, Izabela Chmielewska, Michał Szczyrek, Robert Kieszko, Janusz Milanowski

**Affiliations:** Department of Pneumonology, Oncology and Allergology, Medical University of Lublin, Jaczewskiego 8, 20-954 Lublin, Poland; nataliagalant@umlub.pl (N.G.);

**Keywords:** small-cell lung cancer, SCLC, *MET*, *SLFN11*, chemoimmunotherapy

## Abstract

The possibilities of small-cell lung cancer (SCLC) therapy were strictly limited for years, leading to high patient mortality rates. New approaches to SCLC treatment are being proposed, including chemoimmunotherapy. However, biomarkers enabling appropriate personalization of therapy in SCLC patients have not been identified yet. Even though molecular subtyping (*ASCL1*, *NEUROD1*, *POU2F3*, and *YAP1*) seems pivotal in the management of SCLC, expression of other genes might be potentially valuable during patients’ stratification. Due to their crucial role in tumorigenesis and SCLC invasiveness, benefits arising from *MET* and *SLFN11* gene evaluation are suggested. Our study was designed to evaluate the relationship between the mRNA expression of these genes and chemoimmunotherapy efficacy in SCLC patients. A total of 35 patients with extensive-stage SCLC (ES-SCLC) treated with first-line chemoimmunotherapy were involved in the study. mRNA expression of *MET* and *SLFN11* genes was evaluated using the RT-qPCR technique in FFPE tissue collected from all patients. Molecular results were correlated with clinicopathological features and outcome of disease (OS, PFS). We detected *SLFN11* expression in 60% (21 of 35) of the samples. *SLFN11* expression was higher in patients with longer PFS (*p* = 0.05) and with the T4 feature in the TNM scale (*p* = 0.08). *MET* mRNA was expressed in all FFPE tissues. We observed that risk of progression and death was higher in patients with higher expression of *MET* mRNA (*p* = 0.06 and *p* = 0.04, respectively). Our study showed that *MET* and *SLFN11* expression might serve as additional biomarkers for prediction of chemoimmunotherapy efficacy in ES-SCLC patients.

## 1. Introduction

Small-cell lung cancer accounts for about 15% of all lung cancer (LC) diagnoses. It is a very aggressive disease, characterized by rapid progression. The vast majority of SCLC patients are diagnosed with extensive disease (ED-SCLC), related to poor prognosis, with five-year survival rates of about 10–15% [1].

After decades of being unable to offer patients any treatment other than devastating and often ineffective chemotherapy, currently, the most modern treatment provided to patients with ED-SCLC is immunotherapy in combination with chemotherapy. Its implementation in daily clinical practice is based on the results of the CASPIAN and IMpower133 trials [2,3].

Studies on molecular profiling of small-cell lung cancer (SCLC) have provided much information, which is the starting point for the development of personalized therapies in SCLC. Research showed that it will be possible in the future to use molecularly targeted therapies in patients with SCLC [4,5].

Several molecular subtypes have now been identified that may either discriminate the efficacy of immunotherapy or determine the potential use of molecularly targeted therapies. There are subtypes of SCLC with expression of *ASCL1* (Achaete-Scute Family BHLH Transcription Factor 1), *NEUROD1* (Neuronal Differentiation 1), *POU2F3* (POU Class 2 Homeobox 3), and *YAP1* (Yes1 Associated Transcriptional Regulator) [6,7].

In addition to the expression of four core genes recently described as molecular distinguishers in SCLC, expression of other genes may be biomarkers for personalized treatment efficacy and therapeutic targets. Mentioned in this context are the molecular pathways that are associated with the action of proteins such as MET (MET Proto-Oncogene, Receptor Tyrosine Kinase), ATOH1 (Atonal BHLH Transcription Factor 1), or DLL3 (Delta-Like Canonical Notch Ligand 3) [8,9]. SLFN11 was also included in this group as a potential predictor of patient survival time or response to DNA-damaging drugs [10].

The MET receptor is involved in changes in cell phenotype between epithelial and mesenchymal states, termed the mesenchymal–epithelial transition (MET), which are crucial for the complex remodeling of organ architecture during organogenesis and are considered to promote metastasis of many cancer types. MET is frequently expressed in lung cancer, and its activation is linked to an adverse clinical outcome. MET promotes cell proliferation, invasiveness, and clonogenic growth, contributing to a more aggressive SCLC phenotype [11,12]. This suggests that MET could be a therapeutic target in SCLC [13]. MET overexpression in SCLC has not been studied as thoroughly as in NSCLC, and there is still a gap in this matter. Filling this gap may be particularly important in the face of the emerging trend towards molecular subtyping of SCLC and the introduction of personalized therapies.

SLFN11 is involved in tumorigenesis, inducing replication arrest in the presence of DNA damage through the block of the replication fork [14]. In 196 SCLC tissues, 51% expressed SLFN11, as evaluated by immunohistochemistry (IHC). Further, 69% (20/29) of extra-thoracic high-grade neuroendocrine tumors expressed SLFN11 [4]. SLFN11 is considered a predictive biomarker of the effectiveness of PARP inhibitors in SCLC patients [15]. There is a proposal to use the expression of SLFN11 as a dual biomarker, capturing simultaneously immunological and cancer cell-intrinsic functional dispositions associated with sensitivity to DNA-damaging treatment in high-grade serous ovarian cancer patients [16]. Moreover, a high level of agreement was demonstrated between the expression of mRNA and the expression of SLFN11 protein [17]. In human SCLC cell lines, increased promoter methylation of *SLFN11* has been correlated with resistance to DNA-damaging agents due to low or no *SLFN11* expression [18].

Considering the above, we assessed the expression of *SLFN11* and *MET* genes at the mRNA level. We evaluated the relationship between this expression and the efficacy of chemoimmunotherapy in patients with SCLC.

## 2. Results

**Figure 1:**
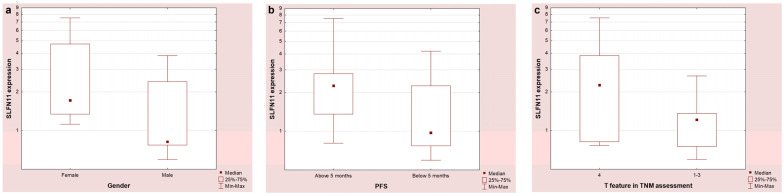
*SLFN11* mRNA expression depending on gender (**a**), duration of PFS (**b**), and T feature on the TNM scale (**c**).

*SLFN11* mRNA expression was identified in 21 patients (60%). Higher expression was observed in females compared to males (Figure 1a, *p* = 0.06), in patients with PFS above 5 months compared to patients with PFS below this value (Figure 1b, 0.05), and in patients with the T4 feature compared to patients with T 1-3 on the TNM scale (Figure 1c, *p* = 0.08). There was also a significant negative correlation between *SLFN11* expression and BMI (R = −0.47, *p* = 0.03). No statistically significant differences were found between *SLFN11* and *MET* expression in patients with partial response, stabilization, or disease progression.

Kaplan–Meier survival analyses were performed for the entire study group. Complete and censored observations for PFS were made in 24 and 11 patients, respectively, and for OS were made in 15 and 20 patients, respectively. For all examined, median progression-free survival was 6 months (95% CI: 4.8 to 6.5) and median overall survival was 12 months (95% CI: 6.2 to 12.5).

**Figure 2:**
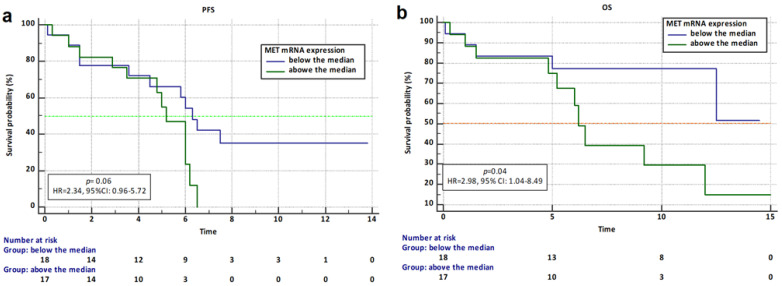
Kaplan–Meier survival curves for PFS (**a**) and OS (**b**) analysis according to the *MET* expression.

The progression-free survival in patients with *MET* expression above the median was observed to be shorter (5.2 months) than in patients with *MET* expression below the median (6.3 months, HR = 2.34, 95% CI: 0.96 to 5.71, *p* = 0.06). (Figure 2a, *p* = 0.06)

The overall survival of patients with higher *MET* expression was shortened (median 6.2 months) compared to patients with lower *MET* expression (median not reached, HR = 2.98, 95% CI: 1.04 to 8.49, *p* = 0.04). (Figure 2b, *p* = 0.04)

## 3. Discussion

The paradigm of SCLC treatment as involving a disease without a targeted treatment option has changed in recent years. The first personalized regimens—including immune checkpoint inhibitors (ICIs), i.e., atezolizumab and durvalumab or the bispecific antibody tarlatamab—were registered by the FDA (Food and Drug Administration) for ES-SCLC treatment in 2019, 2020, and 2024, respectively [19,20]. Following those new possibilities, SCLC has attracted the interest of scientists. Moreover, the implementation of new drugs caused an urgent need to identify biomarkers to enable precise guidance in therapy decision-making. Currently, most of the studies are focused on molecular subtyping of SCLC. However, assessing transcriptional factors overexpressed in specific molecular subtypes may be insufficient for patient stratification [21,22]. Therefore, other biomarkers are being sought. Considering this, we decided to evaluate *MET* and *SLFN11* gene expression at the mRNA level as tools bearing potential predictive value in SCLC patients treated with chemoimmunotherapy.

The *MET* gene is a proto-oncogene encoding one of the tyrosine kinase family receptors. The binding of MET with its principal ligand, HGF (Hepatocyte Growth Factor), leads to the activation of signaling pathways, including RAS-RAF-MEK-ERK and PI3K-AKT [12]. It subsequently affects numerous key cellular functions, such as proliferation, differentiation, growth, and survival [23,24]. Over thirty years ago, Rygaard et al. tested xenografts and cell lines using tissue material collected from 20 SCLC patients. They observed *MET* mRNA expression in 88% of tumors (22 of 25 tumor samples) [25]. Our study detected *MET* mRNA expression in all 35 FFPE samples. However, information regarding the potential role of *MET* assessment in SCLC management is still limited. Wang et al. indicated that *MET* gene is involved in the proliferation and invasion of SCLC and, therefore, that its blockage may reduce the growth of this tumor and its metastatic ability [26]. Moreover, it was proposed that inhibition of MET signaling may be a key to overcoming drug resistance in SCLC patients [27]. Considering the importance of the MET/HGF axis, MET inhibitors were tested in clinical trials in SCLC but unfortunately failed to show efficacy [28]. We suggest that another approach to MET utilization may be more valuable. It has been previously indicated that aberrations in MET signaling may be associated with a worse prognosis in lung cancer patients [29,30], including those with SCLC [11]. We propose *MET* mRNA evaluation as an additional tool enabling more precise prediction in chemoimmunotherapy-treated patients.

Arriola et al. evaluated MET status with the IHC method in 77 tissues collected from SCLC patients treated with chemotherapy. In this study, any MET expression was detected in 75.3% (58 of 77) of samples, while its overexpression was present in 54.5% (42 of 77) tissues. However, the authors do not observe a correlation between MET expression and patient prognosis. OS was associated only with overexpression of p-MET (phosphorylated MET), i.e., activated MET. Overexpression of p-MET was observed in 42.9% (33 of 77) patients and was associated with a worse prognosis (median OS was 132 days in patients with p-MET overexpression vs. 287 days in those with negative or low expression of this protein). Those results suggested that assessment of MET expression in tissue may be insufficient for the prediction of patients’ outcomes and should be expanded with an analysis of its activation. However, in our study, *MET* mRNA expression above the median was associated with a lower median of OS and higher risk of death (*p* = 0.04). A tendency regarding a lower median of PFS and higher risk of progression in the cohort with higher *MET* expression (*p* = 0.06) was also observed. The discrepancy between our results and those obtained by Arriola et al. may be due to the relatively small groups of patients involved in both studies and the implementation of different treatment schemes (chemotherapy vs. chemoimmunotherapy). Moreover, mRNA can be silenced by miRNA, so the protein may not be formed despite its presence. The correlation of *MET* expression at the mRNA and protein levels in the future seems to be valuable.

Miao et al. evaluated MET expression by the IHC method in a slightly larger cohort encompassing 83 SCLC patients. Immunostaining was positive for MET expression in 25.3% (21 of 83) of SCLC cases. The authors presented that the expression of MET was relatively less common compared to other studies. It was indicated that OS is longer in MET-negative patients; however, significance was observed only in patients with a limited stage of disease (median OS was 25.0 vs. 14.0 months; *p* = 0.011). Although simultaneous expression of MET and PD-L1 was detected in 16.9% (14 of 83) of tissues, there was no correlation between PD-L1 (programmed death ligand 1) and MET expression [31]. Assessment of PD-L1 expression on tumor cells is one of the pivotal markers in qualifying NSCLC patients for immunotherapy or chemoimmunotherapy [32]. However, information about the role of PD-L1 expression in the SCLC cohort is currently scarce [31].

Despite MET expression and activation, its mutational status may also be relevant. Bordi et al., in a study encompassing 113 SCLC patients, detected *MET* alteration in 5 cases. However, the authors do not observe any correlation between *MET* mutations and clinical features. OS was similar in patients with *MET*-mutated and wild-type *MET* genes [33]. Voortman et al. pointed out that mutations in the *MET* gene in the SCLC cohort are relatively rare, as they were detected in only 6.5% of clinical samples. Moreover, the authors stated that their presence did not have functional relevance as they did not affect *MET* phosphorylation [34].

*SLFN11* is known to have a multidirectional role in tumorigenesis and to act as a tumor suppressor. It is involved in the inhibition of replication forks in the presence of DNA stress, regulation of chromatin accessibility, and protection against excessive protein aggregation and ubiquitination [14]. It was previously indicated that high expression of *SLFN11* correlated with response to PARP inhibitors (PARPi) and chemotherapy [35,36]. The results of the Raynaud et al. study, conducted on breast cancer cell lines, indicated that restoring SLFN11 activity may be crucial for overcoming chemoresistance [17]. In our study, *SLFN11* expression was detected in 60% (21 of 35) of SCLC FFPE tissues. These results are consistent with the evaluation of SLFN11 expression with the IHC method performed by Qu et al. in 146 primary SCLC samples, as the authors did not observe the expression of SLFN11 in 40% of samples [37].

The study by Qu et al. indicated that the highest SLFN11 histoscore (H-score) was observed in patients with SCLC-Y [37]. As previous studies have pointed out that immunotherapy is the most effective in patients with SCLC-Y or –I subtypes [19], this may simultaneously suggest that higher expression of SLFN11 is associated not only with better response to PARPi and chemotherapy but also with response to ICIs. In our study, higher expression of *SLFN11* was observed in patients with longer PFS compared to patients with shorter PFS. 

Correlation between high *SLFN11* expression and immune activation has been indicated in multiple types of cancer. Isnaldi et al. conducted a meta-analysis that involved 7737 breast cancer cases. The results showed a strong correlation between *SLFN11* and tumor-infiltrating lymphocytes, primarily CD3+ and CD8+ [38]. In the gastric cancer cohort, it was indicated that expression of Schlafen family genes, among others, *SLFN11*, was positively associated with infiltration of numerous immune cells, including CD4+ T cells and CD8+ T cells [39]. Stewart et al. observed that *SLFN11*-high SCLC tumors have an enriched interferon (IFN) signaling pathway and that *SLFN11* expression correlates positively with expression of genes coding immune targets, including PD-L1 (CD274) [40]. It is suggested that higher basal SLFN11 expression could be beneficial during immunotherapy administration. Zhou et al. already proposed a high SLFN11 level in serum as a predictive factor of sensitivity to ICIs in patients with hepatocellular carcinoma [41].

Willis et al. conducted a study that evaluated FFPE tissue collected from 124 SCLC patients. In patients treated with first-line therapy with a combination of platinum with etoposide, longer PFS (median 48.7 vs. 7.8 months) and OS (median 59.7 vs. 13.9 months) were observed in the group with higher SLFN11 H-scores (H-score > 122) compared to patients with lower H-scores. Moreover, high SLFN11 expression was indicated as predictive of either PFS or OS in 45 and 57 patients treated with chemotherapy, respectively [42]. Those results seem to be in concordance with our results, as we observed higher expression of *SLFN11* in patients with median PFS above 5 months (*p* = 0.05). This suggests that *SLFN11* expression may show predictive value in the context of more than one therapeutic regimen. Moreover, as good concordance between mRNA and protein levels of SLFN11 is suspected [17,40], its evaluation on either the genetic or the protein level seems useful.

We observed the tendency between the T feature in the TNM scale and *SLFN11* expression. *SLFN11* expression was higher in patients with T4 than in patients with T1–3 (*p* = 0.08). This may tentatively suggest a potential association between tumor stage and *SLFN11* expression. However, this relation should be analyzed in a larger cohort. Moreover, all of the patients included in our study were diagnosed with ES-SCLC, which is a common situation in this type of cancer. Previously, Masuda et al. reported that higher SLFN11 expression, evaluated with the IHC method, correlated with longer survival. However, this study included only patients with limited-stage SCLC (LS-SCLC) [43]. Willis et al. did not observe a correlation between SCLC stage and SLFN11 protein expression, suggesting that its evaluation does not show prognostic value [42]. As current results regarding the potential predictive role of SLFN11 in SCLC are inconsistent, this topic has to be further assessed. Additionally, reanalyzing using different blocks from the same patient in *SLFN11* and *MET* expression is worth considering when determining the reproducibility and stability of the data. Further, we also observed higher expression (with a tendency towards statistical significance) of *SLFN11* in females compared to males. As there are no specific literature reports related to the differential impact of *SLFN11* on chemoimmunotherapy response based on gender, this result should be verified in an enlarged group of patients.

## 4. Materials and Methods

### 4.1. Studied Group

The study included 35 small-cell lung cancer patients treated with first-line chemoimmunotherapy. Twenty patients were treated with cisplatin and etoposide in combination with durvalumab, and fifteen with carboplatin and etoposide in combination with atezolizumab. All patients were diagnosed with extensive-stage SCLC (ES-SCLC). Characteristics of the study group are shown in Table 1. In all patients, BMI (body mass index) (median = 24.5, min–max: 17.9–37.0, SD = 3.8) and pack years (median = 33, SD = 11.0, min–max: 12–60) were also determined. TNM characteristics were available in 33 (94%) patients. In 10 (30%) patients, the T feature was defined as stages 1–3; in 23 (70%) patients, it was described as stage 4. In 22 (67%) patients, metastases were detected in ipsilateral peribronchial or hilar and ipsilateral mediastinal or subcranial lymph nodes (stages N 1–2). In comparison, 11 (33%) patients presented metastases to contralateral mediastinal or hilar lymph nodes as well as to subclavicular lymph nodes (stage N3). In 7 patients, no distant metastases were found (stage M0), whereas 28 (85%) patients showed distant metastases (stages M1a–M1c).

### 4.2. Gene Expression Analysis

We scored the expression of *SLFN11* and *MET* genes at the mRNA level.

According to the manufacturer’s instructions, the mRNA was isolated from formalin-fixed paraffin-embedded (FFPE) blocks. FFPE blocks were prepared and fixed according to the histopathology department’s routine fixation procedure, using 10% buffered formalin. Paraffin blocks were stored according to the histopathology department’s guidelines. Materials no older than 16 months were used for the study. FFPE paraffin scraps were stored at −20 °C until RNA isolation using the FFPE RNA Purification Kit (EURx, Poland).

RNA quantity and quality were determined using a BioPhotometer (Eppendorf, Germany). RNA was stored at −80 °C until reverse transcription was performed. The High-Capacity cDNA Reverse Transcription Kit (Thermo Fisher Scientific, Waltham, MA, USA) was used according to the manufacturer’s instructions to obtain complementary DNA (cDNA). It was stored at −20 °C until the qPCR (quantitative real-time PCR) reaction was performed.

The qPCR reaction was carried out using probe and primer sets as follows: Hs00536981 (for *SLFN11* mRNA expression examination) and Hs01565584 (for *MET* mRNA expression examination). Internal controls (housekeeping genes) were *GAPDH* (Hs03929097) and *18S rRNA* (Hs03928990). The qPCR reactions were performed on Illumina Eco equipment (Illumina Inc., USA). The reaction mixture consisted of 5 μL of Fast Advanced Master Mix (Thermo Fisher Scientific, USA), 0.5 μL of probe and primer set, 3.5 μL of nuclease-free water, and 1 μL of cDNA. Temperature and time conditions were as follows: initial denaturation at 95 °C for 20 s, then 40 cycles of 95 °C for 3 s and 60 °C for 40 s. Ct analysis was performed using Eco Study software (Illumina Inc., San Diego, CA, USA). The expression was assessed with the use of the 2^−ΔΔCt^ method.

### 4.3. Statistical Analysis

Statistical analysis was performed using Statistica 13.3 (TIBCO Software Inc., Palo Alto, CA, USA) and MedCalc v18.11.6 (MedCalc Software Ltd., Ostend, Belgium). Spearman correlation and the Mann–Whitney U test were used. Kaplan–Meier survival time curves were generated for PFS and OS assessment. A *p*-value of less than 0.05 was accepted as statistically significant, and a 0.09–0.05 range was considered as tending towards statistical significance.

## 5. Conclusions

Although molecular subtyping seems pivotal during SCLC management, additional biomarkers enabling more precise stratification of patients are highly needed. We have evaluated the role of *MET* and *SLFN11* expression at the mRNA level as biomarkers for the prediction of chemoimmunotherapy efficacy in the SCLC cohort. We have observed a relationship between the expression of these genes and clinicopathological features. The relation between *MET* expression and OS was statistically significant, and the association between *MET* expression and PFS showed a tendency towards statistical significance. Moreover, higher *SLFN11* expression was noted in patients with longer PFS. Therefore, we suggest potential benefits from *MET* and *SLFN11* expression evaluation in clinical samples for personalized treatment selection in SCLC patients.

However, it has to be considered that our study is preliminary. It encompassed 35 tissue samples collected from SCLC patients treated with chemoimmunotherapy and needs to be further validated on a larger cohort. Further, in RNA studies, special attention should be paid to the quality of input samples, both paraffin-embedded material and RNA isolates. The tissue fixation process can affect the integrity of nucleic acids. Therefore, it is worth considering analysis using DV200 and RIN (RNA Integrity Number) values when planning to expand the study.

## Figures and Tables

**Table 1:** Characteristics of the studied groups of SCLC patients.

Features	n (%)
**Age (median = 66, SD = 7.4, min–max: 43–80 years)**	
<65 years ≥65 years	16 (46) 19 (54)
**Gender**	
Female Male	17 (49) 18 (51)
**Performance status according to ECOG-WHO**	
0 1	8 (23) 27 (77)
**Bone metastases**	
NO YES	26 (74) 9 (26)
**CNS metastases**	
NO YES	30 (86) 5 (14)
**Liver metastases**	
NO YES	26 (74) 9 (26)
**Treatment-related adverse events (TRAEs)**	
NO YES	17 (49) 38 (51)
**Response to treatment**	
PR SD PD	15 (43) 13 (37) 7 (20)

This study was approved by the Bioethics Committee at the Medical University of Lublin (KE-0254/267/12/2023).

## Data Availability

The original contributions presented in this study are included in the article. Further inquiries can be directed to the corresponding author(s).
